# *Xrcc5/*KU80 is not required for the survival or activation of prophase-arrested oocytes in primordial follicles

**DOI:** 10.3389/fendo.2023.1268009

**Published:** 2023-10-10

**Authors:** Natasha D. Ratnayaka-Gamage, Lauren R. Alesi, Nadeen Zerafa, Jessica M. Stringer, Karla J. Hutt

**Affiliations:** Department of Anatomy and Developmental Biology, Monash Biomedicine Discovery Institute, Monash University, Clayton, VIC, Australia

**Keywords:** DNA repair, DNA damage, oocytes, non-homologous end joining, fertility

## Abstract

**Introduction:**

The non-growing, meiotically-arrested oocytes housed within primordial follicles are exquisitely sensitive to genotoxic insults from endogenous and exogenous sources. Even a single DNA double-strand break (DSB) can trigger oocyte apoptosis, which can lead to accelerated depletion of the ovarian reserve, early loss of fertility and menopause. Therefore, repair of DNA damage is important for preserving the quality of oocytes to sustain fertility across the reproductive lifespan. This study aimed to evaluate the role of KU80 (encoded by the XRCC5 gene) – an essential component of the non-homologous end joining (NHEJ) pathway – in the repair of oocyte DNA DSBs during reproductive ageing, and following insult caused by the DNA-damaging chemotherapies cyclophosphamide and cisplatin.

**Methods:**

To investigate the importance of KU80 following endogenous and exogenous DNA damage, ovaries from conditional oocyte-specific *Xrcc5* knockout (*Xrcc5* cKO) and wildtype (WT) mice that were aged or exposed to DNA damage-inducing chemotherapy were compared. Ovarian follicles and oocytes were quantified, morphologically assessed and analysed via immunohistochemistry for markers of DNA damage and apoptosis. In addition, chemotherapy exposed mice were superovulated, and the numbers and quality of mature metaphase- II (MII) oocytes were assessed.

**Results:**

The number of healthy follicles, atretic (dying) follicles, and corpora lutea were similar in Xrcc5 cKO and WT mice at PN50, PN200 and PN300. Additionally, primordial follicle number and ovulation rates were similar in young adult Xrcc5 cKO and WT mice following treatment with cyclophosphamide (75mg/kg), cisplatin (4mg/kg), or vehicle control (saline). Furthermore, KU80 was not essential for the repair of exogenously induced DNA damage in primordial follicle oocytes.

**Discussion:**

These data indicate that KU80 is not required for maintenance of the ovarian reserve, follicle development, or ovulation during maternal ageing. Similarly, this study also indicates that KU80 is not required for the repair of exogenously induced DSBs in the prophase-arrested oocytes of primordial follicles.

## 1 Introduction

Female mammals are born with a finite supply of non-growing, prophase-arrested oocytes that reside within primordial follicles. Primordial follicle oocytes can remain arrested for up to decades in humans before undergoing folliculogenesis, ovulation and fertilization. During this time, they can be exposed to daily DNA-damaging events as a consequence of endogenous and environmental factors, such as metabolic by-products that accumulate throughout ageing, or chemotherapeutic agents and/or irradiation (1). Primordial follicle oocytes are extremely sensitive to DNA damage, such that even a single double-strand break (DSB) can lead to apoptosis (2). Thus, DNA damage can result in primordial follicle loss and may contribute to the age-associated depletion of the ovarian reserve and reduced oocyte quality. Given that primordial follicles are generated prior to birth and cannot be renewed postnatally, it is crucial to understand how DNA repair can be utilized by oocytes across life to enhance the female fertile lifespan.

There are two pathways a cell can utilize to repair DSBs: homologous recombination (HR) and non-homologous end-joining (NHEJ) (3). HR is a high-fidelity repair pathway that requires an intact DNA sequence to act as a repair template. As such, this pathway is limited to the S and G2 phases of the cell cycle, when a sister chromatid is present (4, 5). In contrast, NHEJ ligates the broken ends of the DNA together and can occur at any stage of the cell cycle, albeit making it an inherently error-prone process (6). Primordial follicle oocytes undergo meiotic arrest after replication of their DNA, and thus sister chromatids are available to facilitate HR-mediated repair. Indeed, recent evidence indicates that HR is preferentially used by primordial follicle oocytes in reproductively young mice to repair DNA DSBs induced by γ-irradiation, while only a small fraction of oocytes will activate NHEJ (7). In contrast, the NHEJ pathway is essential for chromosome integrity in fully grown germinal vesicle (GV) and metaphase II (MII) stage oocytes (8). However, cell cycle checkpoint-mediated death or permanent arrest appears to be reserved for very severe DNA damage in late-stage oocytes (9, 10).

The decline in oocyte number and quality with age is associated with an accumulation of DNA damage in oocytes, in conjunction with a decreased capacity to repair this DNA damage (11). Endogenous damage caused by metabolic by-products – such as reactive oxygen species (ROS) resulting from elevated oxidative stress – increases with age in somatic cells (12). For instance, Titus et al. (11) reported decreased expression in the key HR repair genes *Brca1*, *Mre11*, *Rad51*, and *Atm* in mouse and human GV oocytes with age (11). Another study also reported a significant reduction in BRCA1 function in GV oocytes from older women (13). Interestingly, BRCA1 drives HR-mediated repair by blocking key initiators of NHEJ, including 53BP1 and DNA-PKcs (14). Therefore, the antagonistic actions of BRCA1 on 53BP1 and DNA-PKcs could potentially be alleviated with age, leading to an increased use of NHEJ by oocytes. The notion that NHEJ may play an important role in repairing damage with ageing has been supported by several studies in somatic cells showing that the absence of the NHEJ components DNA-PKcs, KU70, KU80, Artemis, or WRN exacerbates the ageing phenotype in mice and humans (15–18). Additionally, HR efficiency declines with age in human fibroblasts due to impaired binding of RAD51 to damaged DNA, leading to a switch from HR to NHEJ-mediated repair (16). Therefore, it is possible that NHEJ may become the preferential pathway of DNA repair for maintaining the genomic stability of primordial follicle oocytes during ageing, as is the case with somatic cells; though this has not been investigated until now.

X-ray repair cross complimenting 5 (*XRCC5*) encodes the KU80 protein, which dimerises with KU70 to form the DNA-PK heterodimer (7). KU80 is essential for protecting DNA from degradation, and establishing gap filling and ligation, all of which are essential for NHEJ to occur successfully (19). Therefore, NHEJ can be suppressed in oocytes by disrupting *XRCC5* expression. This study aimed to determine the relative importance of KU80 in primordial follicle oocytes for repairing endogenous DNA DSBs during ovarian ageing, as well as exogenously-induced DNA damage. Using wild type (WT) and *Xrcc5* conditional knockout (*Xrcc5* cKO) mice, we show that KU80 – and most likely NHEJ – is not required for the repair of DSBs in primordial follicle oocytes during maternal ageing, or following the induction of DNA damage by cyclophosphamide and cisplatin. These data support prior work which indicates that prophase-arrested primordial follicle oocytes primarily utilize HR to repair DNA damage in the form of DSBs.

## 2 Materials and methods

### 2.1 Mice

Oocyte specific *Xrcc5* conditional knockout mice (*Xrcc5* cKO: Xrcc5^fl/fl^:Tg(Gdf9-iCre) and Cre-negative littermate controls (WT: *Xrcc5* ^fl/fl^:Tg^+/+^) on a C57BL6/J background were generated and validated as described previously (20). Mice were housed in a temperature-controlled high-barrier facility (Monash University Animal Research Laboratory) with free access to mouse chow and water, and a 12-hour light-dark cycle. All animal procedures and experiments were performed in accordance with the National Health and Medical Research Council (NHMRC) Australian Code of Practice for the Care and Use of Animals and approved by the Monash Animal Research Platform Animal Ethics Committee. Genotyping was performed by Transnetyx using real-time PCR.

### 2.2 Tissue collection and processing

To determine the relative importance of oocyte-specific loss of *Xrcc5* on ovarian function with age, ovaries were collected from WT and *Xrcc5* cKO mice at postnatal day (PN) 50 (n=6/genotype), PN200 (n=5-7/genotype), and PN300 (n=7/genotype). To examine whether loss of *Xrcc5* exacerbates the impacts of chemotherapy-induced ovarian damage, WT and *Xrcc5* cKO littermates aged between 7-9 weeks of age were injected intraperitoneally with a single dose of either cyclophosphamide (75mg/kg), cisplatin (4mg/kg), or vehicle control (saline) (21, 22). Animals were allowed to recover for 21 days before superovulation and tissue collection, to allow for primordial follicles to develop to ovulatory oocytes. One ovary from each mouse was fixed in Bouin’s solution for follicle quantification, and the contralateral ovary was fixed in 10% neutral buffered formalin for immunohistochemical analysis.

### 2.3 Follicle quantification

Direct follicle counts were performed to assess any differences in follicle numbers with genotype, age, and/or chemotherapy treatment, as described previously with the following modifications (23, 24). Briefly, Bouin’s fixed ovaries were embedded in glycolmethacrylate resin, serially sectioned at 20µm intervals, and stained with periodic acid Schiff (PAS). Slides were imaged using a Leica Aperio Slide Scanner at 40x magnification and analyzed using ImageScope software. The total number of primordial, primary, secondary, and antral follicles were quantified on every 3^rd^ tissue section, with follicle classes identified according to Myers et al. (23, 25). Raw follicle numbers were multiplied by 3 to account for sections not counted. Follicles were classified separately as atretic (dying) if ≥10% of granulosa cells appeared apoptotic (signified by pyknotic bodies) and/or if intense eosinophilia, zona pellucida degradation and germinal vesical breakdown was observed (26). Additionally, corpora lutea were counted directly by observing each tissue section, to ensure none were double counted.

### 2.4 Immunohistochemistry

Formalin-fixed ovaries were embedded in paraffin and serially sectioned at 5μm intervals. Immunohistochemical analysis for markers of DNA damage (γH2AX) and early-stage apoptosis (cleaved caspase-3 [CC3]) was performed on 4-7 sections per ovary, with at least a 75µm interval between sections to ensure follicles were not double counted (n=4-7 ovaries/genotype/age). Briefly, all slides were dewaxed in histolene and rehydrated in a series of graded ethanols. Heat-induced antigen retrieval was performed in 10mM sodium citrate buffer (pH6) for 10 minutes. Next, endogenous peroxidase activity was quenched using 10% hydrogen peroxide solution (ThermoFisher Scientific) and all washes performed in Tris-NaCl (TN) buffer. All sections were blocked with 10% goat serum (Sigma-Aldrich, G9023) in 3% Bovine Serum Albumin (BSA) (Sigma-Aldrich, A9418) for 30 minutes. Sections were incubated with buffer only (as negative controls) or primary antibodies for 24 hours at 4°C in TN buffer with 1% BSA in the following dilutions: 1/1000 Anti-CC3 (Abcam, ab2302) and 1/500 Phospho-Histone γH2A.X (Ser139) (Cell Signalling Technology, 9718). After washing in Tris-NaCl-Tween (TNT) buffer, slides were incubated with biotinylated goat anti-rabbit secondary antibodies (Vector, BA1000) diluted 1/500, for 1 hour at room temperature. Afterwards, all slides were incubated with avidin-biotin complex Vectastain reagent (Vector Laboratories) for 30 minutes at room temperature. To visualize staining, sections were incubated in 3,3’diaminobenzidine (DAB) chromagen solution (Dako, K3468) for 20-30 seconds. Sections were counterstained with Harris hematoxylin, followed by a quick dip in acid alcohol, and 1 minute in lithium carbonate. Slides were then dehydrated in a series of graded ethanols and histolene, then coverslips were mounted. All slides were viewed under a light microscope (Leica DM500), and the percentage of positively-stained follicles was recorded. Positive follicles were identified by the presence of brown staining in oocyte nuclei and/or in ≥5% of granulosa cells.

### 2.5 TUNEL assay

To analyze follicle atresia, the extent of late-stage apoptosis in each ovary was measured by performing a terminal deoxynucleotidyl transferase (TdT) deoxyuridine triphosphate (dUTP) nick-end labelling (TUNEL) assay utilizing the ApopTag Peroxidase *In Situ* apoptosis detection kit (Chemicon, S7100) following the manufacturer’s instructions. The TUNEL assay was performed on 4-7 paraffinized 5μm sections (n=4-7 ovaries/genotype/age). A positive control section incubated with DNase I was prepared for every run. Apoptotic cells were visualized via the addition of DAB to each section for 5-10 seconds. Subsequently, all slides were counterstained with Harris hematoxylin, followed by a quick dip in acid alcohol, and 1 minute in lithium carbonate. Slides were dehydrated in a series of graded ethanols and histolene, then coverslips were mounted. All slides were viewed under a light microscope, and the percentage of positively stained follicles was recorded. Positive follicles were identified by the presence of brown staining in oocyte nuclei and/or in ≥5% granulosa cells.

### 2.6 Superovulation and oocyte collection

Prior to superovulation, adult (7-9-week-old) WT and *Xrcc5* cKO female mice were injected with a single intraperitoneal dose of cisplatin (4mg/kg), cyclophosphamide (75mg/kg) vehicle control (saline) to induce DNA damage. These doses have previously been shown to induce DNA damage in oocytes without completely depleting the follicle reserve (21, 22). After 21 days, mice were hormone-primed and superovulated with one subcutaneous dose of 10 international units (IU) pregnant mare serum gonadotropin (PMSG), followed 44-48 hours later with 10IU human chorionic gonadotropin (hCG). This timepoint was selected to allow time for primordial follicles to develop into mature, ovulatory oocytes. Cumulus-oocyte-complexes (COCs) were harvested from oviducts 12-14 hours later, and oocytes were denuded using 0.3% hyaluronidase (Sigma-Aldrich) in M2 media. The number of ovulated oocytes of each mouse was recorded, and oocyte cytoplasm, first polar body (IPB), perivitelline space (PVS), zona pellucida, and meiotic spindle were assessed for quality. Observed oocyte morphological abnormalities included increased cytoplasmic granularity, presence of cytoplasmic inclusions, large PVS, PVS granularity, or fragmented IPB. Oocytes were classified as healthy and mature metaphase-II (MII), or poor quality (fragmented/dead). Oocytes were then fixed and permeabilized for 30 minutes in 4% PFA and 2% Triton X-100 and washed in wash buffer (0.1% BSA, 0.1% Tween and 0.01% Triton X-100 in 1x PBS). Oocytes were blocked in 10% BSA and 2% Tween-20 in 1x PBS for 1 hour at room temperature. Subsequently, oocytes were incubated with 1/100 anti-alpha tubulin (Invitrogen, 322588) 1/100 phalloidin (F-actin) (Invitrogen, 872816) and 1/5000 Hoechst (Invitrogen, 879457) in 0.1% BSA in 1x PBS for 60 minutes, then imaged using a Leica SP8 confocal microscope. The proportion of oocytes with normal and abnormal spindles were recorded. Images were analyzed using FIJI software (27).

### 2.7 Statistics

Prior to statistical analysis, data were assessed for normality using a Shapiro-Wilk test. For normally distributed (i.e. parametric) data, unpaired t-tests were performed to compare the differences in follicle and oocyte numbers between two groups, and a one-way ANOVA with Tukey’s *post-hoc* test was performed to compare three or more groups within each age/treatment group. For non-parametric data, a Mann-Whitney U Test was performed to compare two groups, and a Kruskal-Wallis test performed to compare three or more groups. Statistical significance was set at p<0.05.

## 3 Results

### 3.1 Loss of KU80 in oocytes does not deplete the ovarian reserve throughout reproductive life

**Figure 1:**
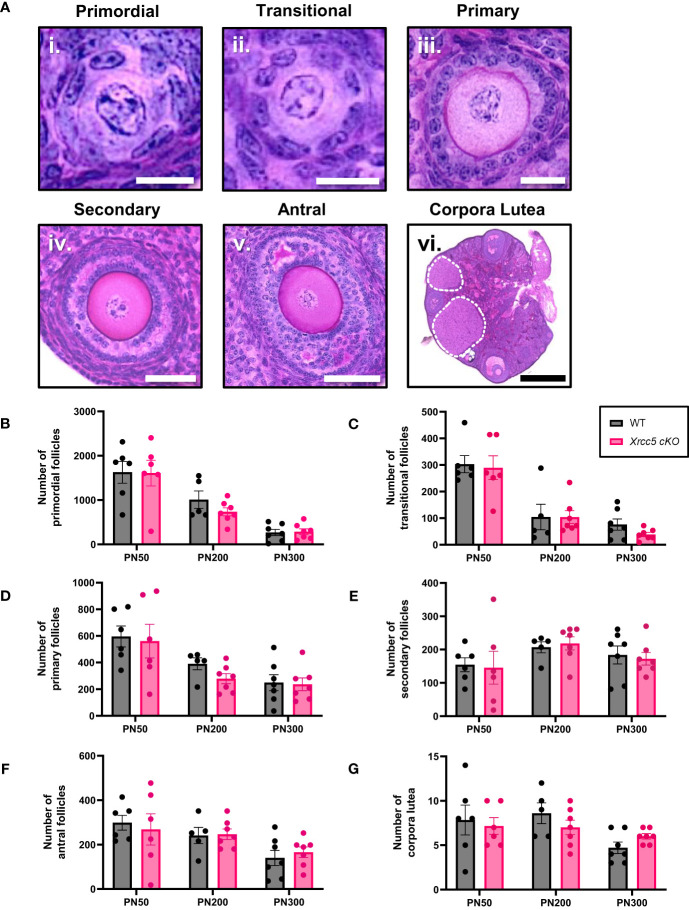
Total number of healthy follicles and corpora lutea recorded in WT and *Xrcc5* cKO mouse ovaries. **(A)** Representative images of healthy i) primordial follicles, ii) transitional follicles, iii) primary follicles, iv) secondary follicles, v) antral follicles, and vi) overview image showing corpora lutea (circled with a dotted line). Scale bar for primordial and transitional follicles = 25µm. Scale bar for primary follicles = 100µm. Scale bar for secondary and antral follicles = 50µm. Scale bar for corpora lutea = 200µm. **(B)** Numbers of healthy primordial follicles in ovaries from WT and *Xrcc5* cKO mice at PN50, PN200, and PN300 (n = 6-7 mice/genotype/age). **(C)** Numbers of healthy transitional follicles in ovaries from WT and *Xrcc5* cKO mice at PN50, PN200, and PN300 (n = 6-7 mice/genotype/age). **(D)** Numbers of healthy primary follicles in ovaries from WT and *Xrcc5* cKO mice at PN50, PN200, and PN300 (n = 6-7 mice/genotype/age). **(E)** Numbers of healthy secondary follicles in ovaries from WT and *Xrcc5* cKO mice at PN50, PN200, and PN300 (n = 6-7 mice/genotype/age). **(F)** Numbers of healthy antral follicles in ovaries from WT and *Xrcc5* cKO mice at PN50, PN200, and PN300 (n = 6-7 mice/genotype/age). **(G)** Corpora lutea quantification in ovaries from WT and *Xrcc5* cKO mice at PN50, PN200, and PN300 (n = 6-7 mice/genotype/age). Data are represented as mean ± SEM and analyzed using unpaired Student’s t-tests.

To determine whether KU80 is essential for maintaining follicle numbers throughout reproductive life, healthy primordial (**Figure 1A**i), transitional (**Figure 1A**ii), primary (**Figure 1A**iii), secondary (**Figure 1A**iv) and antral follicles (**Figure 1A**v), as well as corpora lutea (**Figure 1A**vi) were counted in ovaries from wild type (WT) and *Xrcc5* conditional knockout (*Xrcc5* cKO) mice at PN50 (reproductively young, peak fertility), PN200 (fertile, reproductively aging), and PN300 (reproductively aged, reduced fertility). Ovarian morphology and the number of healthy follicles at each stage of development were similar between WT and *Xrcc5 cKO* mice at each age (**Figures 1B–F**). Additionally, no significant differences were observed in the number of corpora lutea (**Figure 1G**). These data suggest that KU80 is not critical for follicle survival, folliculogenesis or ovulation throughout reproductive life in mice.

### 3.2 Loss of KU80 in oocytes does not increase follicle atresia

**Figure 2:**
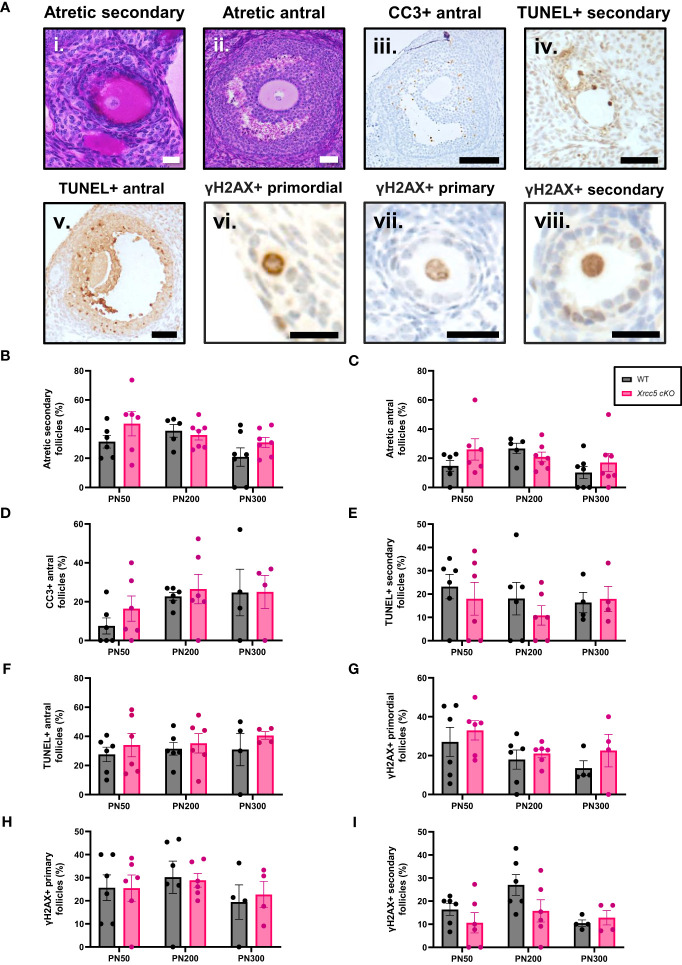
Atretic follicles recorded in WT and *Xrcc5* cKO mouse ovaries. **(A)** Representative images of an i) atretic secondary follicle, ii) atretic antral follicle, iii) cleaved caspase-3 (CC3)-positive antral follicle, iv) TUNEL-positive secondary follicle, v) TUNEL-positive antral follicle, vi) γH2AX-positive primordial follicle, vii) γH2AX-positive primary follicle, viii) γH2AX-positive secondary follicle. Scale bars are equivalent to 50µm for all images excluding γH2AX positive primordial, primary, and secondary follicle images. Scale bars for γH2AX positive primordial, primary, and secondary follicles = 25µm. **(B)** Percentages of atretic secondary follicles observed in WT and *Xrcc5* cKO mice at PN50, PN200, and PN300 (n = 6-7 mice/genotype/age). **(C)** Percentages of atretic antral follicles observed in WT and *Xrcc5* cKO mice at PN50, PN200, and PN300 (n = 6-7 mice/genotype/age). **(D)** Percentages of CC3-positive antral follicles recorded in WT and *Xrcc5* cKO mice at PN50, PN200, and PN300 (n= 4-7 ovaries/genotype/age). **(E)** Percentage of TUNEL-positive secondary follicles observed in WT and *Xrcc5* cKO mice at PN50, PN200, and PN300 (n= 4-7 ovaries/genotype/age). **(F)** Percentage of TUNEL-positive antral follicles observed in WT and *Xrcc5* cKO mice at PN50, PN200, and PN300 (n= 4-7 ovaries/genotype/age). **(G)** Percentages of γH2AX-positive primordial follicles observed in WT and *Xrcc5* cKO mice at PN50, PN200, and PN300 (n= 4-7 ovaries/genotype/age). **(H)** Percentages of γH2AX-positive primary follicles in WT and *Xrcc5* cKO mice at PN50, PN200, and PN300 (n= 4-7 ovaries/genotype/age). **(I)** Percentages of γH2AX-positive secondary follicles in WT and *Xrcc5* cKO mice at PN50, PN200, and PN300 (n= 4-7 ovaries/genotype/age). Between 20-40 follicles were assessed per ovary/genotype/age for CC3, TUNEL and γH2AX staining. All data are represented as mean ± SEM and analysed using unpaired Student’s t-tests.

To determine whether conditional loss of KU80 in oocytes leads to an increase in DNA damage-induced follicle death, atretic (dying) secondary and antral follicles (**Figures 2A**i-ii) were quantified by analysis of morphology. Additionally, the presence of molecular markers of apoptosis (cleaved caspase-3 [CC3] and TUNEL) were also analyzed in secondary and antral follicles (**Figures 2A**iii-v). Neither atretic morphology, nor CC3, or TUNEL staining were detected in any primordial, transitional, and primary follicles, at any age (**Supplementary Figures 1A, B**). While morphologically atretic secondary and antral follicles were observed at all ages, there was no significant difference in the number of atretic growing follicles from WT and *Xrcc5* cKO ovaries (**Figures 2B, C**). Similarly, CC3 was detected in the granulosa cells of antral follicles, and TUNEL staining was detected in the oocytes and/or granulosa cells of secondary and antral follicles in WT and *Xrcc5* cKO. However, there were no significant differences in the proportions of CC3- and TUNEL-positive follicles between the genotypes at PN50, PN200 or PN300 (**Figures 2D–F**). These data suggest that loss of KU80 in oocytes does not increase secondary or antral follicle atresia, regardless of age.

### 3.3 Loss of KU80 in oocytes does not lead to accumulation of endogenous DNA damage with age

To determine if DNA damage accumulates in the absence of KU80, γH2AX (DSB marker) staining was assessed in PN50, PN200 and PN300 ovaries. Positive γH2AX staining was observed in the nucleus of oocytes and granulosa cells of primordial, primary, and secondary follicles (**Figures 2A**vi-viii), but not antral follicles (**Supplementary Figure 1C**). There was no significant difference in the proportion of γH2AX-positive follicles in ovaries from WT and *Xrcc5* cKO mice at any age (**Figures 2G–I**), suggesting that loss of KU80 in oocytes does not lead to the accumulation of endogenous DNA damage across the fertile lifespan.

### 3.4 Loss of KU80 in oocytes does not sensitize oocytes to DNA damage

**Figure 3:**
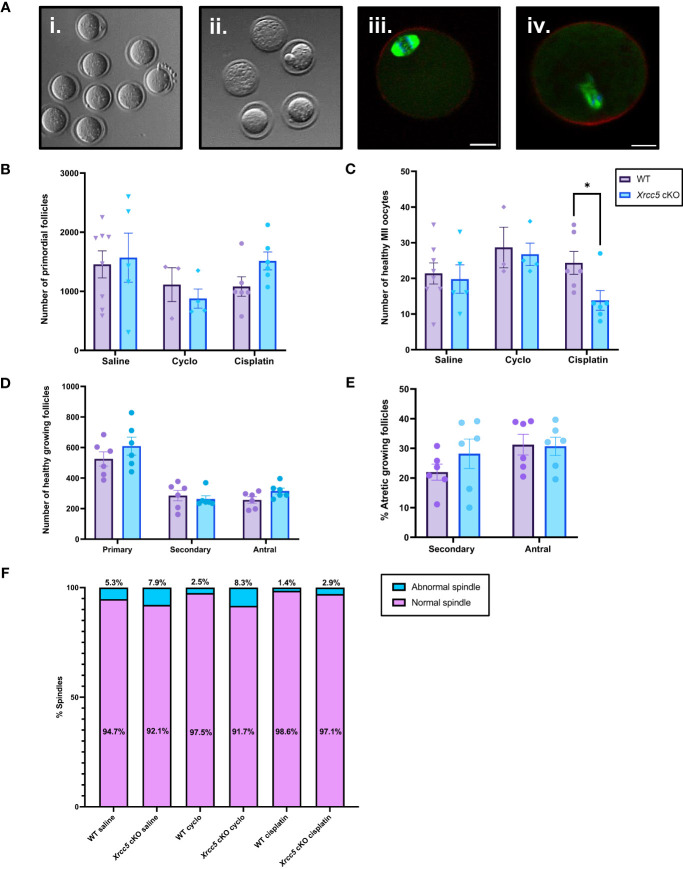
Healthy MII and poor-quality oocytes ovulated from WT and *Xrcc5* cKO mice treated with cyclophosphamide, cisplatin, or vehicle control. **(A)** Stereoscopic images of ovulated oocytes morphologically classed as either i) healthy MII, or ii) poor-quality. Representative images of morphologically classified healthy MII oocytes with iii) normal and iv) abnormal spindles. Actin was stained with phalloidin (red), DNA was stained with Hoechst (blue), and mitotic spindles were stained with anti-tubulin (green). Scale bars = 20µm. **(B)** Primordial follicle number in WT and *Xrcc5* cKO mice treated with either 75mg/kg cyclophosphamide, 4mg/kg cisplatin, or vehicle control (saline). **(C)** Healthy MII oocyte numbers in WT and *Xrcc5* cKO mice treated with 75mg/kg cyclophosphamide, 4mg/kg cisplatin, or vehicle control (saline). **(D)** Numbers of healthy primary, secondary, and antral follicles in WT and *Xrcc5* ckO mice treated with 4mg/kg cisplatin. **(E)** Percentages of atretic secondary and antral follicles in WT and *Xrcc5* cKO mice treated with 4mg/kg cisplatin. **(F)** Proportions of morphologically healthy MII oocytes presenting with normal and abnormal spindles, for each group. Data are represented as mean ± SEM, n= 3-8 mice/genotype/treatment, analyzed using a one-way ANOVA, followed by a *post-hoc* Tukey’s multiple comparisons test **(B, C, F)** or an unpaired t-test **(D, E)**, * = p < 0.05.

We next sought to determine if oocyte-specific *Xrcc5* deletion increases the sensitivity of primordial follicles to DNA-damaging agents, or reduces the yield or quality of ovulated follicles. Young adult WT and *Xrcc5* cKO mice were treated with a single dose of cyclophosphamide, cisplatin, or vehicle control (saline), sufficient to induce DNA damage in oocytes without ablating the ovarian reserve (21, 22). After 21 days, mice were superovulated by exogenous hormonal stimulation. Oocytes were collected, quantified and classified as either healthy MII (**Figure 3A**i) or poor-quality (fragmented/dead; **Figure 3A**ii). Immunostaining was then performed to visualize meiotic spindles, which were classified as either normal (**Figure 3A**iii) or abnormal (**Figure 3A**iv). Ovaries were also collected for follicle quantification.

Primordial follicle loss was not exacerbated in *Xrcc5* cKO mice compared to WT mice following treatment with cyclophosphamide or cisplatin (**Figure 3B**). Healthy MII oocyte yield was similar in WT and *Xrcc5* cKO animals treated with saline or cyclophosphamide (**Figure 3C**). Interestingly, however, there was a significant reduction in the number of healthy MII oocytes collected from cisplatin-treated *Xrcc5* cKO mice compared to cisplatin-treated WT mice (WT 24 ± 3.2 vs *Xrcc5* cKO 14 ± 2.8, p = 0.0323) (**Figure 3C**). The number of healthy primordial, primary, secondary, and antral growing follicles did not significantly differ between WT and *Xrcc5* cKO cisplatin treated mice (**Figure 3D**). Similarly, the percentage of atretic secondary and antral follicles did not significantly differ between either genotype of cisplatin-treated mice (**Figure 3E**). These data suggest that loss of KU80 does not significantly alter the rate of secondary or antral follicle development or atresia, following cisplatin-induced DNA damage. Thus, the reduction in oocyte yield cannot be explained by a reduction in the number of oocytes available for ovulation. Further analysis confirmed that most oocytes morphologically classified as healthy MII stage had normal spindles (bipolar spindle, focused poles, aligned DNA) and there were no differences in the proportion of oocytes with normal and abnormal spindles within each treatment group (**Figure 3F**).

## 4 Discussion

In this study, we show that KU80 is dispensable for primordial follicle survival and follicle development during maternal aging, when endogenous DNA damage accumulates as a consequence of normal metabolic processes (11, 28). Similarly, KU80 is not required for primordial follicle survival following the induction of exogenous DNA damage by cyclophosphamide or cisplatin. Together, these observations suggest that NHEJ-mediated repair may not be essential for oocytes at this stage of development. This is consistent with previous studies, which suggest that HR is the most prominent DNA repair pathway utilized by primordial follicle oocytes (7, 29).

Some studies have suggested that oocytes in growing follicles are more resistant to DNA damage, and are thus less likely to undergo apoptosis than the non-growing oocytes in primordial follicles (28). One explanation for this is that apoptotic mechanisms may be more tightly regulated during the oocyte growth phase. Alternatively, it is possible that once activated to begin growth, oocytes rely more heavily on the DNA repair response than apoptosis to ensure oocyte integrity. This makes sense, as oocyte growth coincides with dramatic increases in gene transcription, protein production, and cellular metabolism processes that increase the likelihood of endogenously-induced DNA damage (30, 31). Interestingly, in this study, loss of KU80 within the oocytes of growing follicles did not result in accumulation of unrepaired DNA damage or increased follicle atresia at any age. These data indicate that DNA DSBs that arise in growing oocytes as a consequence of normal cellular activities are effectively repaired in the absence of KU80, and suggest that NHEJ may not play an essential role in oocyte survival during folliculogenesis.

Primordial follicle loss after exposure to cyclophosphamide and cisplatin was not exacerbated by loss of KU80, suggesting that KU80 is not required for oocyte survival after the induction of DNA damage. However, it is possible that in the absence of KU80, the surviving oocytes harbored unrepaired DNA damage, compromising their ability to undergo meiotic maturation and ovulate. Indeed, 21 days after cisplatin treatment, the number of healthy MII oocytes retrieved after hormonal priming was significantly lower in *Xrcc5* cKO mice than WT mice, although the number of abnormal oocytes was not significantly increased. We have recently shown that in fully grown germinal vesicle-stage oocytes, the NHEJ pathway is important for the repair of exogenously induced DSBs and chromosome integrity during oocyte maturation (20). Further analysis of the growing follicle population in this study showed no significant difference in the number of healthy growing follicles in WT and *Xrcc5* cKO mice treated with cisplatin. Additionally, the proportion of atretic growing follicles did not significantly differ between WT and *Xrcc5* cKO mice. These data could indicate that a small proportion of KU80-deficient oocytes with irreparable DNA damage underwent atresia prior to ovulation to reduce the number of healthy MII oocytes ovulated and/or explain the absence of an increase in poor quality oocytes ovulated.

Interestingly, it is worth noting that normal numbers of healthy MII oocytes were ovulated from *Xrcc5* cKO mice following cyclophosphamide treatment. The reason for this difference between cisplatin and cyclophosphamide is unclear, but it could relate to the slightly different modes of action of these drugs, or possibly the differential efficacy of the corresponding DNA repair pathways (32). For instance, cyclophosphamide is cleaved to hydroxycyclophosphamide, which is metabolized to aldophosphamide, which is cleaved to form the alkylating agent phosphoramide mustard (33). The phosphoramide mainly acts by cross-linking DNA which may prevent strand separation, block DNA replication and/or DNA transcription, which may contribute to the accumulation of DNA DSBs (34). This form of DNA damage may be repaired by proteins encoded by Fanconia anemia (FA) genes, many of which are HR repair genes (35). Indeed, it has been well-established that mutations in FA genes lead to increased sensitivity to DNA alkylating agents (34, 36). Therefore, it is possible that the DNA damage induced by cyclophosphamide may have been repaired by genes involved in the HR pathway, which was functional in this study. Cisplatin predominantly operates by covalently binding to DNA bases to cause mono-, inter- and intra-strand adducts, though to a lesser extent they can also induce inter-strand cross-links (37–40). Similar to cyclophosphamide, this interferes with DNA replication machinery by blocking transcription and translation, which ultimately leads to the formation of DSBs (37, 38). While HR and NHEJ repair is essential for repairing cisplatin-induced DNA double strand breaks (29, 41–43), the DNA adducts are predominantly repaired by nucleotide excision repair (NER) pathways, such as transcription-coupled repair (TCR) or global repair (GR) (38). Interestingly, one study demonstrated that DNA-PK-deficient mouse somatic cells were sensitized to cisplatin and UV-C irradiation, resulting from reduced NER activity (44). Whilst it has not been established that KU80 or DNA-PK are physically incorporated in the NER process, these components may still be important for NER activation and/or progression. Therefore, the indirect inhibition of crucial DNA repair pathways (such as NER) through KU80 deletion may contribute to the reduction in ovulated oocyte numbers following cisplatin treatment in *Xrcc5* cKO mice. Although unclear, the contrasting number of healthy MII oocytes ovulated by KU80 deficient mice treated with cyclophosphamide and cisplatin may be explained by the differing efficacy of alternative DNA repair pathways utilized to repair the DNA damage.

In conclusion, our data suggest that KU80 is not essential for the repair of endogenously-induced double strand breaks in primordial follicle oocytes, or following exogenously induced DNA damage. This study provides novel evidence to support the hypothesis that HR is the main DNA repair pathway utilized by primordial follicle oocytes to repair DNA double-strand breaks.

## Data availability statement

The raw data supporting the conclusions of this article will be made available by the authors, without undue reservation.

## Ethics statement

The animal study was approved by Monash University Animal Ethics Committee. The study was conducted in accordance with the local legislation and institutional requirements.

## Author contributions

NDR-G: Investigation, Formal Analysis, Project Administration, Visualization, Writing – original draft, Writing – review & editing. LA: Investigation, Methodology, Visualization, Writing – review & editing. NZ: Investigation, Project Administration, Writing – review & editing. JS: Investigation, Conceptualization, Formal Analysis, Funding acquisition, Methodology, Project Administration, Supervision, Visualization, Writing – review & editing, Writing – original draft. KH: Conceptualization, Formal Analysis, Funding acquisition, Methodology, Resources, Supervision, Writing – original draft, Writing – review & editing.

## Supplementary material

Supplementary Figure 1 Control images obtained for CC3 and γH2AX immunohistochemical staining, and TUNEL assays. **(A)** Representative positive and negative control images for cleaved caspase-3 (CC3) immunohistochemical staining. Scale bars = 200µm. **(B)** Representative positive and negative control images for TUNEL staining. Scale bars = 50µm. **(C)** Representative positive and negative control images for γH2AX immunohistochemical staining. Scale bars = 200µm. For all experiments, a positive control was included for each run, and a negative control was included for each slide.
