# Supplementary figures and images for: Xrcc5/KU80 is not required for the survival or activation of prophase-arrested oocytes in primordial follicles
Source: Front Endocrinol (Lausanne). 2023 Oct 10;14:1268009. doi: 10.3389/fendo.2023.1268009 (PMC10603181; doi:10.3389/fendo.2023.1268009)

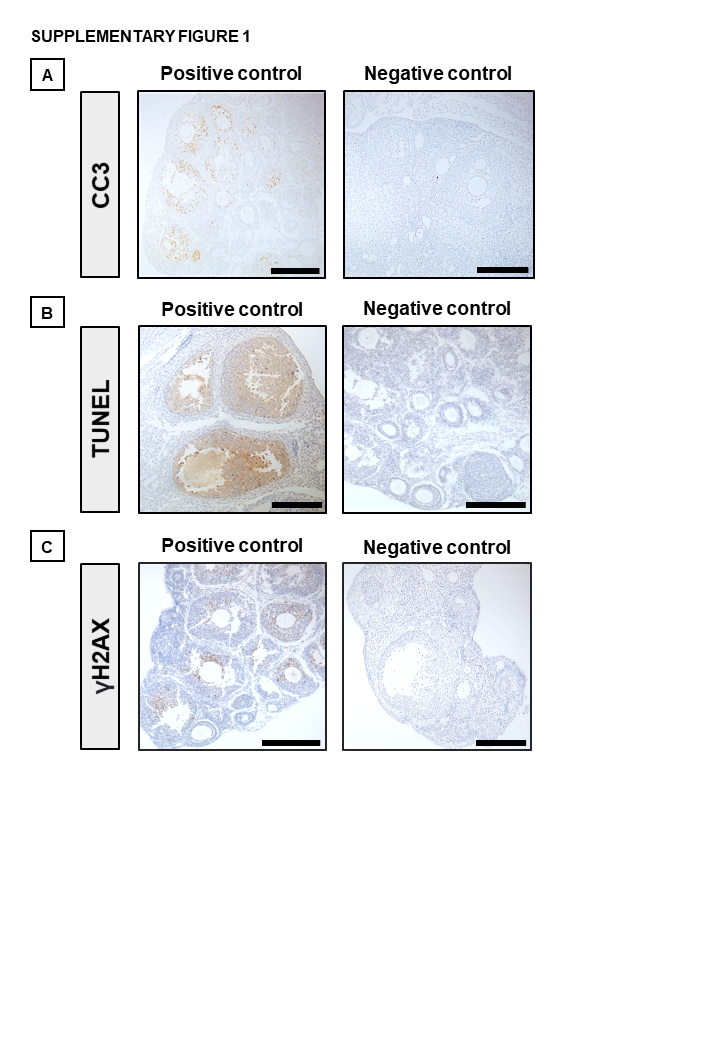

Supplement: Supplementary Figure 1 — Control images obtained for CC3 and γH2AX immunohistochemical staining, and TUNEL assays. (A) Representative positive and negative control images for cleaved caspase-3 (CC3) immunohistochemical staining. Scale bars = 200µm. (B) Representative positive and negative control images for TUNEL staining. Scale bars = 50µm. (C) Representative positive and negative control images for γH2AX immunohistochemical staining. Scale bars = 200µm. For all experiments, a positive control was included for each run, and a negative control was included for each slide. [file Image_1.tif]
